# Female Education

**Published:** 1856-09

**Authors:** A. P. Merrill


### *Female Education.* By A. P. Merrill, M. D.

There are few subjects of public interest, which present stronger claims upon the consideration, not only of medical men, but of the community generally, than the circumstances which by a law of common consent, attach themselves to the discipline and instruction of females. With commendable zeal and liberality the people of the South-western States have devoted a vast amount of money, and a large show of talent and learning, to the business of educating and training young females. In view of their own future happiness, and the important station which they are to hold in reference to the present and future generations, too high an estimate is not likely to be attached to this great interest; nor are we to apprehend, that too large a portion of the wealth and talent of the country will ever be appropriated to its support. But there may be danger, that, in the great efforts now making to impart useful knowledge to females, together with those habits of study and reflection, which result from early mental discipline, and the refining accomplishments, music, painting, &c., without corresponding arrangements for an equivalent

† Rokitansky's Pathological Anatamy, vol. 3, p. 275.

system of instruction for the male sex, a discrepancy may be created between those two branches of the rising generation, the tendency of which will be to unfit them for mutual and reciprocal harmony in taste and feeling, and thus lay the foundation for ill-assorted matches, and much of discontent and unhappiness in their domestic relations.

This aspect of the subject commends itself to the consideration of the moralist, and may not be unworthy the regard of the statesman and patriot; but it is to the physiological and hygienic questions that we propose more particularly to address ourselves on this occasion, and we hope to be able to present this branch of the subject in such a manner as to engage the attention of physicians. It will scarcely be questioned that it is not more the business and province of enlightened and liberal physicians, to study the best means for the relief and cure of diseases already established than to discover and make known measures of prevention, and the requisites for improving and amending the physical constitution. If it can be made to appear as true, or even if any well grounded apprehension can be established, that the liberal efforts now making to cultivate the understanding of females, are attended in most cases by such conditions as must necessarily lead to a deterioration of constitutional vigor, and the establishment of feeble health, and feeble bodily powers, it will behoove the medical profession, aided as they may be by the parents of the rising generation, to concert measures of relief—measures for the remedy of an evil of so serious a character as to threaten the well-being, happiness, and even the matured existence of that portion of our race, which is so truly esteemed as *God's last best work.*

No fact is better established than that the powers of mind and body are always impaired by excessive, and especially by unequal use. Persons of all ages are liable to become enfeebled by either excessive idleness or excessive labor, while a just medium will as certainly secure a healthy performance of all the bodily functions, resulting in stability and strength, and by consequence, comfort and happiness. And so, also, of the mental faculties, exercise, even to a laborious extent, is a condition of acknowledged necessity to the existence of mental health and vigor, and even to mental soundness. Inactivity of the mind, long continued and habitual, leads with great certainty in the downward direct tion of feeble-mindedness and even idiocy, while the opposite condition, long continued over-exertion, without proper periods of rest and recuperation, leads just as certainly in the direction of mental unsoundness and insanity. Both conditions by their extremes, and, by destroying that equilibrium between mental and bodily vigor, which nature intended should exist, have the effect, too often observed among us, to impair the healthy functions of the body, and thus to shorten life.

Now, we are forced to believe, and must, therefore, contend, however unpalatable and unpopular it may be in certain quarters, that the systems of school discipline which so generally obtain in female academies are such as must inevitably lead to the impairment and deterioration of both bodily and mental health. Girls are placed in these institutions either just before, or, in a majority of cases, just after they have arrived at the age of puberty—a period of life when the whole nervous system is peculiarly impression, when the digestive organs are active, gorged with blood and liable to feverish excitement, and when the brain and spinal marrow are in progress of rapid development, and exceedingly tender.

These are conditions which require active, regular and almost constant muscular exercise, without exhausting fatigue, abundance of nutritious and digestible food, joyous mental diversion, sound and refreshing sleep, and the careful avoidance of a long continued burden upon the spinal column and pressure upon the viscera of the abdomen and pelvis. It is important, also, that each individual should enjoy, at all times, a full supply of fresh and uncontaminated air, and that the extremities should not be benumbed with cold, or the circulation in them impeded by exhausting muscular inaction, or by pressure of the limbs across the edge of high seats.

But what is the course commonly pursued with pupils in these female schools? The account we have received from an intelligent young lady under our care for the relief of serious ill-health contracted in one of these schools, substantiated as it is by information derived from various other sources, may be taken as a tolerably correct type of a large majority of the female academies in the South-west. We are aware of some exceptions to the rigid confinement and extraordinary requirements, as indicated in this narrative, but are quite convinced that the ameliorations are not generally approved by the parents of the pupils, many of whom seem to think they are not requited with the worth of their money, unless their children are engaged in study during all the day and a portion of the night. This feature exonerates from blame, to some extent, the managers of schools; for, unless their arrangements are made acceptable to their patrons, their pupils will certainly be withdrawn; and this fixes the responsibility where it properly belongs, with parents and guardians. Parents are constantly making the discovery, when it is too late to remedy the evil, that the health and happiness of their daughters have been sacrificed, and that young women born to the inheritance of a sound constitution, and a life of independence and plenty, have become even before attaining their full growth, the pitiable victims of irremediable disease, which is rendered the more painful and distressing, in many cases, by the constant but ineffectual efforts which are made to obtain relief. Victims in the first place to a false system of mental and bodily training, they become the painful and tantalized victims to the various phases of charlatanry which prevail, and sometimes die in the very prime of life, of some one of those chronic diseases which should only be found the accompaniment of mature age and decay.

The narrative of a pupil, to which we refer, is in effect as follows: We are required to rise with the sun, and in winter somewhat earlier. Only a few minutes are allowed for dressing, when we are summoned to prayers. These being ended a number of the girls practice their music, while those without instruments study their lessons. An hour is thus spent, when breakfast is announced. We proceed to the table and eat quickly and silently. A half hour or so is then allowed for rest and recreation, but no active and boisterous exercises are permitted. Then we enter the school room and submit to a strict confinement for four hours, relieved only by a recess of fifteen minutes. While not reciting lessons it is deemed a serious infraction of rules, to look up from our books. The first session is ended by the signal for dinner, which is a short and frugal meal, and those whose turns to practice their music come after dinner, are obliged to hurry away to their tasks. The afternoon session begins after the intermission of an hour, and continues three hours, with one recess of fifteen minutes. Then a portion of the pupils are dismissed, a few take their turns practicing their music, and nearly always a considerable number are detained in school, because of their not having given satisfaction, either in their lessons or behavior. After an early supper, an hour in winter, and an hour and a half in summer, is spent in silent study, when the signal is given to return to bed. Much of the time in the winter season we sit in a room which is so imperfectly warmed that our feet are benumbed with cold, and to prevent a still greater depression of temperature, ventilation is carefully guarded against. The air of our crowded dormitories is rendered particularly impure by confinement, which is painfully apparent to those who enter them from the fresh air.

We had designed introducing here a memorandum furnished by an experienced teacher, but the account given in this differs very little from the above, showing fully as much time devoted to study and close confinement. It represents also, that in some boarding schools so little time is allowed for eating that the food is too rapidly consumed, and when the signal is given for retiring from the table some are still eating. "The whole arrangement for eating was as unphysiological as it well could be. The food was not in quality or quantity suited to the wants of the young ladies. The principal meat was fat bacon—sometimes boiled with greens—but often fried and swimming in fat, which for lack of butter was eaten with avidity by the hungry pupils. Dishes of fat, greasy gravy were a prominent feature on the table."

There is, of course, not a perfect uniformity in the arrangements of different schools; but all are objectionable in a greater or less degree, in the confinement and want of exercise, in the long and continued mental application, in the multiplicity of duties and studies, in the want of ventilation and warmth, in the diet and rapidity of eating, and in the want of joyous mental recreation. The mind of the pupil is treated as a safe and capacious receptacle for all sorts of knowledge, which has only to be thrown in pell-mell, and all being retained without wastage, for future use, the subject is well educated just in proportion to the variety and quantity supplied; while the bodily frame is considered in the light of one of those ingeniously contrived engines, which only requires to be wound up every morning, and well greased three times a day, to be kept running perpetually, and without change.

Now, the effect of all this upon the constitution and functions of both body and mind, may readily be divined by every well informed and experienced physician The mind, crowded with a medley of strange facts and theories which it has not time to arrange or digest, and habituated to learn without habits of thought and reflection, presents a confusion of ideas which can never be reduced to order, and causes a surfeit of study which can never be overcome. And this is called a *finished* education. More palpable still is the impression made upon the bodily organization and functions. The digestion is impaired, the secretions are deranged, and the nervous system has lost its healthy tone and vigor. Constipation, habitual headache, depraved appetite, disturbed sleep, menstrual obstruction, swollen extremities, fatigue upon slight exertion, cough, frequent alternations of mental excitement and depression, and sundry and various anomalous derangements of the bodily functions, render the girl sickly, unhappy, and ill-tempered, at the very period of life when she should and might be in the full and happy enjoyment of both bodily and mental health, buoyant and lively, at peace with herself and all the world besides. We do not mean to say that all suffer to the same extent, but none can fail to experience some of these ill effects, if subjected to such discipline for any length of time; and many there are, doubtless, who continue to suffer through life, and transmit the effects of their unphysiological education to succeeding generations.

The evils of injudicious systems of youthful training, are rifest in those portions of our country where there has been the least experience. Time and observation correct them. Take the ancient city of Boston as an example. There was a period when the evils we complain of existed there to the same extent they do now here. Constitutions destroyed, and the hopes of parents blighted, have drawn to the subject the attention of the ablest and most influential men, under whose influence and advice such reformations have been effected, as would seem to an inexperienced observer to indicate the existence of an opposite extreme. Witness the following regulation of the "*Girl's High and Normal School*:"

"There shall be one session of five hours each day, from 8 A. M. to 1 P. M., from May to October, and from 9 A. M. to 2 P. M., from October to May."

In this and all other schools in Boston a recess is not only allowed for recreation, but it is provided that every pupil shall have daily some kind of physical or gymnastic exercise, and the following regulation is also rigidly enforced:

"In assigning lessons to be studied out of school hours, the instructors shall not assign a larger lesson daily, than a boy of good capacity can acquire by an hour's study; but no out of school lessons shall be assigned to girls, nor shall the lessons to be studied in school be so large as to require a scholar of ordinary capacity to study out of school in order to learn them."

These regulations result from two centuries' experience in the instruction of youth, and in a city which has produced and is now producing annually, a larger number of scholars, male and female, of high educational attainments, than any other city in the world. The present aim and purpose is, to rival all other cities of the world, also, in the constitutional health and mental vigor of its people, of both sexes.

We call upon the educated men and women of this part of the country; who, from their own experience and their knowledge of the opinions of others, are qualified to judge of the importance of a reformation of the evils complained of, to set about effecting a change in public opinion, upon which alone can the reformation be founded. And we call especially upon physicians, who witness daily in the most difficult and uncontrollable diseases, the result of these errors in mental education and bodily training, to come to the rescue, and make such efforts as they can to preserve this interesting portion of our population from constitutional ruin, and from the frightful prevalence of those diseases peculiar to the sex, which are among the *opprobria* of our profession.

The evils of excessive confinement, interminable study, multiplicity of books and superficial teaching, resulting mainly from attempting to do too much in a given period of time, extend to all southern schools, so far as we have observed them. Besides the monstrous error before referred to, that education is successful in proportion to the length of time allotted to study, and the variety of branches taught, there is another error scarcely less mischievous prevailing, which considers it a part of the province of schools, to act the part of nurseries, to keep young children out of the streets and out of mischief, and thus to relieve parents from a considerable portion of their onerous duties, to the sacrifice of health, and the impairment of the usefulness of teachers. Public sentiment has done and is doing much to reform these evils, and in those parts of our country where they enjoy the benefit of the best experience, five hours a day is the longest time allowed for school exercises, and there are those who contend that four and even three hours are sufficient for this purpose. *Memphis Medical Recorder.*

